# Autochthonous Lactic Acid Bacteria Isolated From Dairy Cow Feces Exhibiting Promising Probiotic Properties and *in vitro* Antibacterial Activity Against Foodborne Pathogens in Cattle

**DOI:** 10.3389/fvets.2020.00239

**Published:** 2020-05-15

**Authors:** Wen-Chin Lin, Christopher P. Ptak, Chi-Yu Chang, Man-Kei Ian, Min-Yuan Chia, Ter-Hsin Chen, Chih-Jung Kuo

**Affiliations:** ^1^Department of Veterinary Medicine, National Chung Hsing University, Taichung, Taiwan; ^2^Institute of Preventive Medicine, National Defense Medical Center, Taipei, Taiwan; ^3^NMR Facility, Roy J. and Lucille A. Carver College of Medicine, University of Iowa, Iowa City, IA, United States; ^4^Graduate Institute of Veterinary Pathobiology, National Chung Hsing University, Taichung, Taiwan

**Keywords:** probiotics, *Lactobacillus*, microbiota, antimicrobial activity, species specificity

## Abstract

Bovine enteric bacterial pathogens are a major cause of health decline in agricultural cattle populations. The identification of host-derived microbiota with probiotic characteristics is key for the development of treatments utilizing pathogen displacement and recolonization by commensal flora. In this study, intestinal microbiota in fecal samples from four Holstein dairy cows were analyzed using 16S ribosomal RNA gene next-generation sequencing, leading to the identification of three *Lactobacillus* isolates (*Lactobacillus gasseri, Lactobacillus reuteri*, and *Lactobacillus salivarius*). By taking advantage of the preferential growth in acidified culture media, bacterial characteristics examination, and restriction fragment length polymorphism analysis of 16S rRNA genes, the three lactic acid bacteria (LAB) strains were successfully isolated. The three LAB isolates possess the prerequisite growth tolerances for probiotic functionality, as well as exhibit effective antimicrobial potency against enteric bacterial pathogens of cattle, including *Escherichia coli* O157:H7, *Mycobacterium avium* subspecies *paratuberculosis*, and *Salmonella* species (*Salmonella enteritidis, Salmonella typhimurium*, and *Salmonella* Dublin). Moreover, the LAB isolates showed significant adhesion to cattle intestine, implying greater survivability potential due to their species specificity when administered in the same host species. The LAB isolates were sensitive to most antibiotics with notable resistances of *L. gasseri* to streptomycin and *L. salivarius* to kanamycin. Genes attributed to specific antibiotic resistances demonstrated a low risk of lateral transfer in a conjugation study. Our *in vitro* results demonstrate the promising probiotic characteristics of these newly identified *Lactobacillus* strains and their considerable potential to serve as probiotics feed supplements for cows.

## Introduction

Probiotics are live microorganisms that benefit humans and animals by promoting intestinal tract health. The major beneficial attributes of probiotics include the ability to compete with enteric pathogens, the contribution to increased digestive capacity, the enhancement of mucosal immunity, and the reduction of intestinal pH. As a result of these characteristics, probiotics create an unfavorable environment for the growth of enteric pathogens and act to prevent disease-causing bacteria from colonizing the intestine. Probiotic bacteria reduce a host's inflammatory responses by stabilizing the gut microbial environment, fortifying the intestinal permeability barrier, facilitating the degradation of enteral antigens, and altering their antigenicity and immunogenicity (1). Accordingly, the natural gut microflora is more stable and resilient to population disruptions. Several probiotic strains (*Bifidobacterium* species, *Lactobacillus* species, *Saccharomyces boulardii*, and *Streptococcus thermophilus*) have been effectively utilized for young cattle with positive results on overall health, feed efficiency, weight gain, and immunocompetence (2).

One of the most prominent commensal flora used as probiotics belong to the genera, *Lactobacillus*, commonly referred to as lactic acid bacteria (LAB). Lactic acid bacteria are characterized as Gram-positive, rod-shaped, acid-tolerant, facultative anaerobic or microaerophilic, fermentative, and non-respiring. The effectiveness of LAB as a probiotic agent is linked to an ability to survive in stomach acid and bile salt (BS) and to adhere to and colonize the intestinal lining (3). Like many probiotic bacteria, LAB exhibit either bactericidal or bacteriostatic properties. Direct antimicrobial activity by *Lactobacillus* species is derived from the production of organic acids, hydrogen peroxide, bacteriocins, and low-molecular-weight compounds (4). Lactic acid bacteria isolates from dairy cow fecal samples have been shown to possess both the survivability and antimicrobial properties of effective probiotics (5). In swine, LAB isolates can preferentially serve as probiotics for the originating host animal, suggesting a role for diet and species specificity in probiotic efficacy (6). The goal of probiotic treatment is to colonize the lower intestine of young preruminants with healthy flora, such as LAB, thereby facilitating the maintenance of the gut microbiota and the elimination of enteric pathogens. Because probiotic bacteria might adhere to specific epithelial cells of the intestine, an isolate from the host itself will be more adaptable to support a commensal relationship with the host as compared to bacteria from dissimilar organisms.

Bovine gut infections by enteric pathogens lead to huge economic losses resulting from higher rates of culled cattle, reduced reproductive and feed efficiency, and poor milk production or low-quality products (7). Foodborne pathogens, such as *Escherichia coli* O157:H7 and *Salmonella* species, can be detected on the hides or in the gastrointestinal tract (GIT) of meat animals. Ruminants can also spread the disease by acting as asymptomatic natural reservoirs while shedding pathogens in feces (8). Supportive treatments for effectively limiting the course and severity of disease include fluid supplementation with electrolytes, non-steroidal anti-inflammatory drugs, and probiotics. Another agriculturally important enteric pathogen, *Mycobacterium avium* subspecies *paratuberculosis* (MAP) is both the causative agent of Johne disease (JD) affecting ruminant species and the etiologic agent of Crohn disease, an inflammatory bowel disease in humans. Currently, no effective vaccines or medications are available for MAP-related diseases. In recent years, treatment options have examined the use of probiotics against *Mycobacterium* species and other enteric pathogens as a possible strategy for the displacement of disease-causing gut microbiota from ruminants (9, 10).

In this study, fecal bacterial microbiota of four healthy dairy cows were analyzed by next-generation sequencing technology and found to contain three *Lactobacillus* strains, including *Lactobacillus gasseri, Lactobacillus reuteri*, and *Lactobacillus salivarius*. The identified *Lactobacillus* strains were further isolated through screens for growth on acidified media [MRS (de Man, Rogosa, and Sharpe) at pH 5.4], biochemical identification (Gram stain and catalase test), and restriction fragment length polymorphism (RFLP). In addition to exhibiting good probiotic properties and digestive enzyme activities, three *Lactobacillus* isolates also possess both *in vitro* antimicrobial activity against several enteric bacterial pathogens and species-specific binding to bovine intestinal cells. Despite resistances to streptomycin and kanamycin were, respectively, detected in *L. gasseri* and *L. salivarius*, the newly identified *Lactobacillus* strains were assessed to have a minimal risk of lateral antibiotic-resistance gene transfer. These *Lactobacillus* strains isolated from the native host may serve as potential probiotic candidates for cattle and further support a native probiotic-to-host strategy.

## Materials and Methods

### Fecal Sample Collection and DNA Extraction

Four Holstein dairy cows were selected at the National Chung Hsing University located in subtropical Taiwan from October 2018 to January 2019 (detailed information in Table 1). Cows were housed, fed, and monitored under the supervision of Dr. Jacky Peng-Wen Chan as previously described (11). Briefly, feedings were provided twice daily with a diet of total mixed ration, whereas fresh water was provided freely. Fecal samples were collected from the rectum with gloves and 220-mg and 1-g pieces were weighted for subsequent genomic DNA extraction and bacteria isolation, respectively. Genomic DNA extraction was performed by using QIAamp DNA Stool Mini Kit (QIAGEN, Hilden, Germany). Purified DNA concentration was determined via UV absorption using a Nanodrop spectrophotometer (Nanodrop 1000; Thermo Fisher Scientific, Wilmington, MA, USA). DNA samples that gave an A260/280 ratio between 2 and 1.8 were stored at −80°C until further processing.

**Table 1:** Four Holstein dairy cows used in this study.

**Cow ID**	**400**	**413**	**414**	**427**
Breed	Holstein			
Parity	2	1	1	1
Body condition score^a^	3.0	2.75	3.0	3.0
Average daily milk yield (kg)	26	28	27	26
*M. bovis*^b^	Negative			
*M. avium* subspecies *paratuberculosis*^c^	Negative			

a *The body condition scores were evaluated by a 5-point body condition scoring system*.

Diagnosis of M. bovis and M. avium subs. paratuberculosis was performed by serological examination using

b IDEXX M. bovis Ab test and

c *paratuberculosis screening Ab test ELISA kits*.

### Fecal Microbiota Analysis Using an Illumina MiSeq Platform

The V3–V4 regions of the bacteria 16S rRNA gene were amplified by polymerase chain reaction (PCR) using primers 338F (5′-barcode- ACTCCTRCGGGAGGCAGCAG)-3′ and 806R (5′-GGACTACCVGGGTATCTAAT-3′) with a unique eight-base “barcode” sequence. The PCR program consisted of an initial denaturation step at 95°C for 2 min, followed by 25 cycles at 95°C for 30 s, 55°C for 30 s, 72°C for 30 s, and a final extension step at 72°C for 5 min. Triplicate PCR reactions were performed in a 20-μL mixture containing 4 μL of 5 × FastPfu buffer, 2 μL of 2.5 mM dNTPs, 0.8 μL of each primer (5 μM), 0.4 μL of FastPfu DNA polymerase, and 10 ng of template DNA. Amplicons were purified using the Qubit dsDNA HS Assay Kit (Thermo Fisher Scientific) and quantified by a Qubit 2.0 Fluorometer (Thermo Fisher Scientific). Amplicons were paired-end sequenced (2 × 250) on an Illumina MiSeq platform. Final 16S rRNA sequences were classified by phylogenetic affiliation to a confidence threshold of 70% as previously described (12). The coverage percentage was concluded using the MOTHUR program (http://www.mothur.org), and the raw pyrosequencing reads were submitted to Sequencing Read Archive (SRA) database under the accession id: SRA061866. Genera operational taxonomic units (OTUs) with relative abundances >0.05% of the total bacteria were designated as predominant genera OTUs and sorted for cross group comparisons. The raw data was shown in Supplementary Table 2.

### Bacterial Isolation and Identification

**Figure 1:**
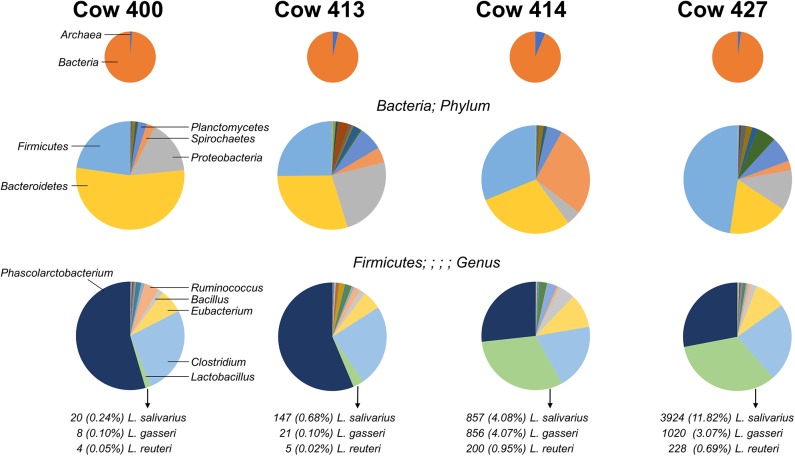
Distribution of microbes identified from four cow fecal samples. Pie charts indicate the organism distribution at the kingdom level **(Top)**, the bacteria distribution at the phylum level **(Middle)**, and the Firmicutes distribution at the genus level **(Bottom)**. The chart data are arranged from largest to smallest count for the combined total of the four cows, and the highest-ranking members are noted (left charts). The count and percentage of isolated *Lactobacillus* species are listed.

The fecal sample from cow 414 was selected for the isolation of *Lactobacillus* bacteria because it possessed both robust *Lactobacillus* colonization and the most homogeneous *Lactobacillus* species distribution (Figure 1). Fecal samples (1 g) collected from cow 414 were homogeneously mixed with 9 mL of sterile phosphate-buffered saline (PBS) by a vortex mixer. Serial 10-fold dilutions were carried out and plated in 0.5-mL aliquots onto de Man, Rogosa, and Sharpe (MRS; BD Difco, Franklin Lakes, NJ, USA) agar. Initial attempts to isolate *Lactobacillus* strains utilized MRS agar plates at pH 6.5 but yielded colonies with a large variety of different morphological features. In order to inhibit the growth of non-acidophilic bacteria, the fecal sample of cow 414 was homogeneously mixed serial 10-fold dilutions plated on acidified MRS agar (pH 5.4). Plates were incubated at 37°C for 48 h under anaerobic condition (AnaeroPack; Mitsubishi Gas Chemical, Tokyo, Japan). Colonies with transparent halo-surrounding characteristics were subcultured in acidified MRS broth (pH 5.4). This procedure was repeated in order to obtain single colonies. From the acidified plates, approximately 80 colonies with creamy white features were observed. For the isolation of LAB, colonies were further examined by Gram staining and catalase production. Gram staining was performed by using the Difco/BBL Gram stain kit (BD, Franklin Lakes, NJ, USA). Smears were observed using a light microscope (ZEISS Primo Star, Carl Zeiss Microscopy GmbH, Jena, Germany) under oil immersion. For catalase tests, 1 drop of 3% hydrogen peroxide (H_2_O_2_) was placed onto the microorganism on the microscope slide. Positive reactions were evident by immediate effervescence. Isolate selection was based on strict criteria, where only rod-shaped Gram-positive and catalase-negative isolates were included. All isolates were identified to be *Lactobacillus* from tests for Gram staining and catalase activity. Pure, original cultures were stored at −80°C in MRS broth containing 50% glycerol. Only 43 isolates that exhibited the coccibacilli, Gram-positive, catalase-negative, and creamy white colonies features were finally picked up for further examination using the RFLP analysis of 16S rDNA genes. The amplification of 16S rDNA was using the universal primers 8F (5′-AGAGTTTGATCCTGGCTCAG-3′) and 1541R (5′-AAGGAGGTGATCCAGCCGCA-3′). The PCR reaction mixture (100 μL) was composed of 50 μL Taq DNA Polymerase 2 × Master Mix RED (Ampliqon, Odense, Denmark), 50 ng of bacterial genomic DNA, 0.4 μM of each primer (8F and 1541R), and the genomic DNA extracted from each isolate (50 ng). The PCR was run for 35 cycles using the following conditions: 3 min at 94°C of initial denaturation, 35 cycles consisting of 15 s at 96°C, 90 s at 60°C, and 90 s at 72°C and a final extension at 72°C for 7 min. The PCR products were purified using the QIAquick PCR Purification Kit (QIAGEN).

### RFLP Analysis of 16S rDNA

The 16S rDNA sequences of the three *Lactobacillus* strains (*L. gasseri, L. reuteri*, and *L. salivarius*) identified in the fecal samples were obtained from the database of the National Center for Biotechnology Information (NCBI, http://blast.ncbi.nlm.nih.gov), and determination of suitable restriction endonucleases for RFLP analysis was performed by a virtual restriction digest analysis tool (RestrictionMapper version 3, http://www.restrictionmapper.org). Three restriction endonucleases, including *Eco*RI, *Sna*BI, and *Nco*I, were selected for *Lactobacillus* typing. Polymerase chain reaction–amplified 16S rDNA (480 ng) of each isolate was digested with individual *Eco*RI, *Sna*BI, or *Nco*I (New England Biolabs, Beverly, MA, USA) at 37°C for 20 h in 15 μL volumes with recommended reaction buffers. Digested 16S rDNA products were electrophoresed in 2% agarose gels (10 × 10 cm) at 100 V for 75 min. The gels were stained with ethidium bromide and visualized by an UV transilluminator. The final confirmation of the isolates was achieved by the 16S rDNA sequence analysis.

### Growth Rate and Resistance to Simulated Gastric and Small Intestine Juices

To monitor growth of three isolates, 0.5 μL of each isolate (OD_600_ at 0.6) was cultured in 5 mL MRS broth (pH 6.5) and maintained at 37°C anaerobically. Bacterial colony-forming units (CFUs) were counted after plating serial dilutions on MRS agar plates to determine the bacterial concentration of the inoculum. Simulated gastric juice was prepared by dissolving 3 g/L pepsin (Sigma-Aldrich, Taufkirchen, Germany) in salt solution (125 mM NaCl, 7 mM KCl, and 45 mM NaHCO_3_) and pH adjusted to 3.0 by 0.1 N HCl. Preparation of simulated small intestinal juice was performed by adding 0.15% BS (Sigma-Aldrich, St. Louis, MO, USA) and 0.1% pancreatin (Sigma-Aldrich) in salt solution (45 mM NaCl) and pH adjusted to 8.0 by 0.1 M NaOH. Both solutions were prepared freshly and sterilized through a 0.22-μm filter membrane. Three isolated strains were inoculated in 5 mL of MRS broth (pH 6.5) and incubated at 37°C for 48 h under anaerobic condition, and then 1 mL culture was used for viable count on MRS agar at pH 6.0 (control). For resistance to gastric tolerance test, two vials (1 mL in each vial) of overnight culture were separately inoculated in 5 mL of simulated gastric (pH 3.0) at 37°C for 1.5 h. Cells were then harvested by centrifugation (2,600 × g at 4°C). The pellet of one vial was resuspended in sterile 0.85% NaCl and plated on MRS agar. Remaining pellet was subsequently inoculated to 5 mL small intestinal juices (pH 8.0) at 37°C for 5 h. Cells were processed following the same procedure as mentioned above and plated on MRS agar. MRS agar plates were incubated anaerobically at 37°C for 48 h. Percentage of resistance (or survival rate) to gastric and small intestinal juices was determined by counting the number of colonies on MRS plates using the following equation:

$$\begin{array}{l} \text{Survival rate \%}=\frac{100\times \text{CF}{\text{U}}_{\text{pH}3.0\text{ or pH}8.0}}{\text{CF}{\text{U}}_{\text{pH}6.0}} \end{array}$$

### Autoaggregation and Hydrophobicity Assay

Bacteria were grown in MRS broth for 18 h at 37°C. After centrifugation at 5,000 × g for 15 min, cells were washed twice and resuspended in filter-sterilized PBS (pH 7.3) to give viable counts of ~10^8^ CFUs/mL. To mix, 4 mL of the cell suspension was vortexed for 10 s. Autoaggregation measurements were conducted after 2-h incubation at 37°C. The upper suspension (100 μL) was transferred to a separate tube containing 3.9 mL of PBS. The absorbance (600 nm) of the mixture was measured and autoaggregation was calculated according to the equation:

$$\begin{array}{l} 1-\left(A/A0\right)\times 100 \end{array}$$

where *A* represents the absorbance after 2-h incubation, and *A*0 is the absorbance before incubation. For hydrophobicity tests, bacterial strains were grown in MRS broth with cysteine at 37°C for 24 h. After centrifugation at 5,000 × g for 15 min, the pellets were washed twice with PBS as described previously. Bacterial suspension (2 mL) was transferred into another tube, and 0.4 mL of xylene was added (Sigma-Aldrich). Tubes were shaken for 2 min and reposed for 15 min, and the absorbance (600 nm) of the aqueous phase was measured. A decrease in aqueous phase absorbance was to determine cell surface hydrophobicity (%*H*). %*H* was calculated according to the following equation:

$$\begin{array}{l} \left[\left(A0-A\right)/A0\right]\times \;100 \end{array}$$

where *A*0 is the absorbance before xylene extraction, and *A* is the absorbance after xylene extraction.

### Digestive Enzyme Activity Assay

Lactic acid bacteria isolate enzymatic activities were evaluated using a spot inoculation method on enzyme-specific agar medium (13). To determine LAB strain amylase, lipase, and phytase activities, the *Lactobacillus* isolates were selected and grown in MRS broth. Bacterial culture (50 μL) was transferred onto a disc (disc diameter 5 mm, thickness 0.10 mm) placed over relevant agar medium and incubated anaerobically for 48 h at 37°C. After incubation, the diameter of halo zone surrounding each colony was measured. For amylase activity detection, a medium consisting of meat peptone (0.5%), yeast extract (0.7%), NaCl (0.2%), starch (2%), and agar (1.5%) was used. Gram iodine served as a detecting agent to facilitate the observation of clear zones. Lipase activity was detected by using medium consisting of tryptone (0.1%), yeast extract (0.5%), NaCl (0.05%), olive oil (1%), Arabic gum (1%), and agar (1.5%). Medium consisting of glucose (1.5%), calcium phytate (0.5%), NH_4_NO_3_ (0.5%), KCl (0.05%), MgSO_4_ (0.05%), MnSO_4_ (0.02%), FeSO_4_ (0.001%), and agar (1.5%) at pH 7.0 was used for phytase activity detection. For protease activity, a medium consisting of skim milk (1%) and agar (1.5%) was used to detect the clear zones surrounding each disc.

### *Ex vivo* Adhesion to Intestinal Epithelium

Intestinal samples from cattle (2 years old) and chick (6 weeks old) were generously provided by Dr. Chia-Lin Ho (The Animal Disease Diagnostic Center, National Chung Hsing University, Taichung, Taiwan). Reference *Lactobacillus* strains, including *Lactobacillus ruminis* [Bioresource Collection and Research Center (BCRC) 14620^T^] isolated from bovine rumen and *Lactobacillus aviarius* subspecies *aviaries* (BCRC 14048^T^) isolated from chicken feces, were purchased from the BCRC (Hsinchu, Taiwan). To loosen the surface mucus, the tissues were soaked in PBS at 4°C for 30 min followed by three successive PBS washes. A LAB isolate suspension (10^9^ cells/mL PBS) was incubated with intestinal tissue (1 × 1 cm^2^) at 37°C for 30 min and then gently washed with PBS to remove unbound bacteria for slice preparation and adhered *Lactobacillus* quantification. Samples were first fixed in 10% formalin, then dehydrated with increasing ethanol concentrations, and finally embedded in paraffin. Serial sections were cut to a width of 5 μm and assessed, respectively, by Gram stains. In addition, *Lactobacillus*-bound intestinal samples were washed by vortex in a 1.5-mL Eppendorf with PBS (1 mL). The supernatant was collected after centrifugation (1,000 × g for 1 min). Bacterial cells were stained by methylene blue and counted by the Neubauer chamber (Celeromics, Grenoble, France) using a light microscope.

### Antibiotic Susceptibility Testing and Minimum Inhibitory Concentration Determination

The minimum inhibitory concentration (MIC) for each antibiotic was determined using the broth microdilution method in the MRS broth in 96-well cell culture plates (Thermo Fisher Scientific). Antibiotics, including ampicillin, vancomycin, gentamicin, kanamycin, streptomycin, erythromycin, clindamycin, tetracycline, and chloramphenicol (Sigma-Aldrich), were diluted in MRS broth and tested in a series of twofold dilutions at the concentration range of 0.12 to 256 μg/mL. Wells were inoculated with 200 μL of the bacterial culture (1.5 × 10^7^ CFUs/mL) and incubated at 37°C anaerobically. Each antibiotic's MIC was read after 24 h of incubation to determine the lowest antibiotic concentration for which growth was visibly inhibited. Bacteria cutoff values and antibiotics resistance profiles were determined according to the guidance of the European Food Safety Authority (14).

### Detection and Transferability of Resistance Genes

Bacterial genomic DNA was isolated as mentioned in *Fecal Sample Collection and DNA Extraction*. The presence of genes conferring resistance for streptomycin and kanamycin was determined by PCR (15). Antibiotic resistance gene-specific primers are listed in Table 2. The PCR reaction was carried out and analyzed following the same procedure described above. Transferability of antibiotic-resistance genes was determined using a slightly modified version of the filter mating method described previously (16). Briefly, the donor (*L. gasseri* and *L. salivarius*) and recipient (*Enterococcus faecalis* JH2-2) strains were, respectively, grown in non-selective broth to reach an OD_600_ of 0.5. Equal amounts of the donor (1 mL) and the recipient (1 mL) bacterial cultures were mixed and collected on a membrane filter with 0.45-μm pore size (HAWP02500; Millipore, Billerica, MA, USA). The membrane was then incubated overnight on brain heart infusion (BHI) agar (BD, Sparks, MD, USA) at 37°C. Membrane was washed by BHI medium (2 mL) to collect the bacteria. Mating mixture dilutions were spread on BHI agar plates containing either streptomycin (100 μg/mL)/rifampicin (50 μg/mL) or kanamycin (50 μg/mL)/rifampicin (50 μg/mL) at 37°C for 48 h. A clinically isolated *Salmonella choleraesuis* (04-1087) (17) harboring *tet*(M) gene (tetracycline resistance) was used as a reference (donor) strain. All matings were repeated three times in duplicate.

**Table 2:** Primers used for PCR detection of antibiotic-resistance genes.

**Antibiotic**	**Antibiotic resistance gene**	**Primer (5^**′**^-3^**′**^)**	**Amplicon size (bp)^a^**
Streptomycin	*aad*A	CCTCGTGTAATTCATGTTCTGGC ATCCTTCGGCGCGATTTTG	282
	*aad*E	GCAGCGCAATGACATTCTTG ATGGAATTATTCCCACCTGA	565
	*ant(6)*	TCAAAACCCCTATTAAAGCC ACTGGCTTAATCAATTTGGG	597
Kanamycin	*aph(3″)-III*	GCCTTTCCGCCACCTCACCG GCCGATGTGGATTGCGAAAA	292
	*ant(2″)-I*	GCTTGATCCCCAGTAAGTCA GGGCGCGTCATGGAGGAGTT	329

a *Ouoba et al. (15)*.

### Antagonistic Activity Against Bovine Enteric Pathogens

The effects of cell-free supernatants (CFSs) secreted by *Lactobacillus* on the growth of *E. coli* O157:H7, *Salmonella enteritidis, Salmonella typhimurium, Salmonella* Dublin were evaluated using the Oxford Cup method (18). *Lactobacillus* isolate was cultured in 100 mL MRS broth at 37°C for 24 h. For CFS collection, cell growths were centrifugation at 10,000 × g for 10 min, and the supernatant was sterilized by filtration using syringe filters (0.45-μm Millipore membrane; Millipore). Briefly, each pathogen (the number of cells in McFarland 0.5) was coated on trypticase soy agar solid medium at 1:100 (vol/vol) with the Oxford Cup (inner diameter = 6 mm, outer diameter = 8 mm, height = 10 mm). Cell-free supernatants of *Lactobacillus* isolates (100 and 200 μL) were added at the midpoint of the Oxford Cup. The diameter of the clear zone around each Oxford Cup was measured after incubation at 37°C for 24 h. The equal volume of acidified MRS broth (pH 4.0) was used as a reference. Inhibition zones for each group were measured three times. In addition, MAP K-10 strain (ATCC, Manassas, VA, USA) was grown in Middlebrook 7H9 broth of pH 6.6 (BD, Sparks, MD, USA) supplemented with 0.24% glycerol, 0.05% Tween 80, 1.2 mg/mL casitone (BD, Franklin Lakes, NJ, USA), 1.25 mg/L mycobactin J (Allied Monitor, Inc., Fayette, MO, USA), and 10% Middlebrook oleic albumin dextrose catalase enrichment (BD, Franklin Lakes, NJ, USA) at 37°C in T75 tissue culture flasks. Bacterial cells were centrifuged for 10 min at 1,500 × g. After washing with PBS, the harvested cells were resuspended in a PBS buffer volume to reach an OD_600_ of 0.9. For anti-MAP assay, 100 μL of MAP culture (OD_600_ at 0.9) was inoculated with 4.5 mL 7H9 broth and 0.5 mL CFSs of *Lactobacillus* isolate or MRS (control) in a T25 flask at 37°C. *Mycobacterium avium* subspecies *paratuberculosis* cells were stained by methylene blue and counted by the Neubauer chamber (Celeromics) at day 28 using a light microscope. All experiments were repeated three times, and the mean bacteria number was calculated from the three repeated data.

## Results

### Analysis of the Fecal Microbiota by Next-Generation Sequencing of the 16S rRNA Gene

The V3–V4 regions of the bacteria 16S rRNA extracted from healthy cow rectal samples were subjected to an Illumina MiSeq Platform for sequencing. The four cows exhibit a diversity of intestinal flora (Figure 1), with the most prominent bacterial phyla varying greatly between individual rectal samples. The distribution for prevalent Firmicutes present with two major patterns as cows 400 and 413 had high (>50%) *Phascolarctobacterium* and low (<5%) *Lactobacillus*, and cows 414 and 427 had similar levels (~30%) *Phascolarctobacterium* and *Lactobacillus*. Three *Lactobacillus* species (*L. gasseri, L. reuteri*, and *L. salivarius*) were identified from the fecal samples with varied percentage. Because the fecal sample from cow 414 had both robust *Lactobacillus* colonization and the most homogeneous *Lactobacillus* species distribution (Figure 1), *Lactobacillus* bacteria for further characterization were isolated from the cow 414 sample.

### 16S rDNA-RFLP Analysis of *Lactobacillus* Isolates

**Figure 2:**
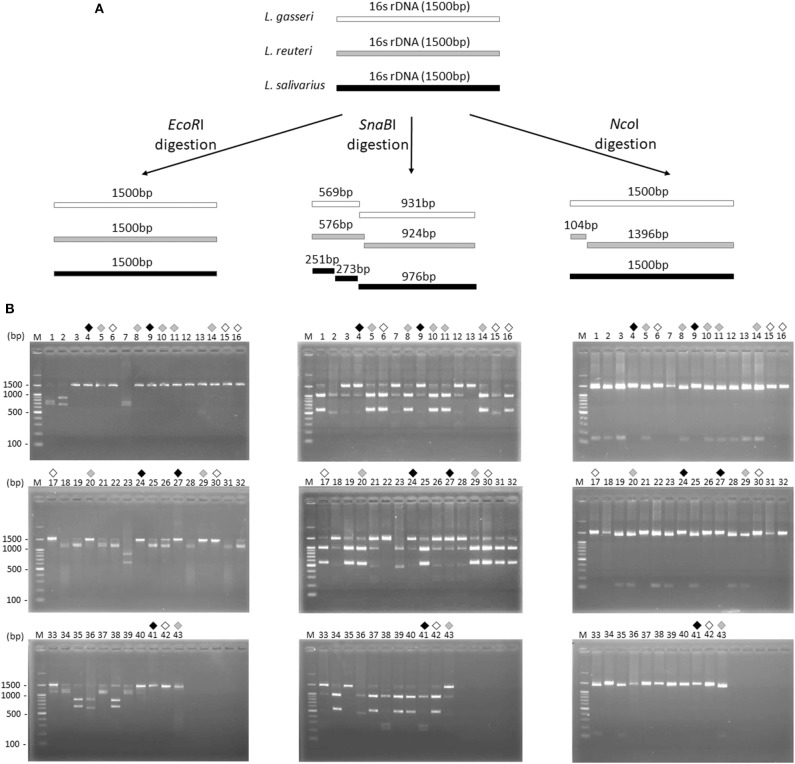
Restriction fragment length polymorphism analysis of 16S ribosomal DNA of three *Lactobacillus* species. **(A)** Illustration of the 16S rDNA fingerprint patterns of *L. gasseri* (white bar), *L. reuteri* (gray bar), and *L. salivarius* (dark gray bar) after *Eco*RI, *Sna*BI, and *Nco*I digestion, respectively. **(B)** The 16S rDNA fingerprint patterns digested with *EcoR*I (left panel), *Sna*BI (middle panel), and *Nco*I (right panel) of 43 colonies isolated from MRS agar plates. Suspected colonies of *L. gasseri* (black open diamond), *L. reuteri* (gray diamond), and *L. salivarius* (black diamond) were indicated.

16S rDNA sequence of *L. gasseri, L. reuteri*, and *L. salivarius* (obtained from NCBI database) were analyzed by a virtual restriction digest analysis tool (RestrictionMapper version 3). As shown in Figure 2A, *Eco*RI is a noncutting restriction enzyme for the 16S rDNAs of three isolates. *Sna*BI cleaves *L. gasseri* and *L. reuteri* 16S rDNAs once, generating DNA fragments of 569/931 and 576/924 bp, respectively. *Sna*BI cleaves 16S rDNA of *L. salivarius* twice, generating DNA fragments of 251/273/976 bp *Nco*I cuts 16S rDNAs of *L. reuteri* at one site, producing DNA fragments of 104/1396 bp but serves as a non-cutting restriction enzyme for the 16S rDNAs of *L. gasseri* and *L. salivarius*. For actual 16S rDNA digestion (Figure 2B), 16S rDNAs of 43 isolates were amplified by PCR and then digested individually by using *Eco*RI, *Sna*BI, or *Nco*I. Colony 6, 15, 16, 17, 30, and 42 (black open diamond) maintained intact length of 16S rDNA (1,500 bp) after *Eco*RI and *Nco*I digestion but generated the fragment sizes of 569/931 bp after *Sna*BI digestion, which were corresponded with the virtual restriction patterns of *L. gasseri*. Colony 5, 8, 10, 11, 14, 20, 29, and 43 (gray diamond) exhibited intact length of 16S rDNAs (1,500 bp) after *Eco*RI digestion and the fragment sizes of 596/924 bp and 104/1396 bp after digestion with *Sna*BI or *Nco*I, respectively, representing the expected restriction profiles of *L. reuteri*. The restriction profiles of colony 4, 9, 24, 27, and 41 (black diamond) showed the undigested 16S rDNAs (1,500 bp) after treating with *Eco*RI and *Nco*I and displayed fragment sizes at 251, 273, and 976 bp, which were suspected to be the strain of *L. salivarius*. The final confirmation of candidate colonies of *L. gasseri, L. reuteri*, or *L. salivarius* was achieved by the 16S rDNA sequence analysis (Supplementary Table 1).

### Characteristics and Probiotic Properties of *Lactobacillus* Isolates

**Figure 3:**
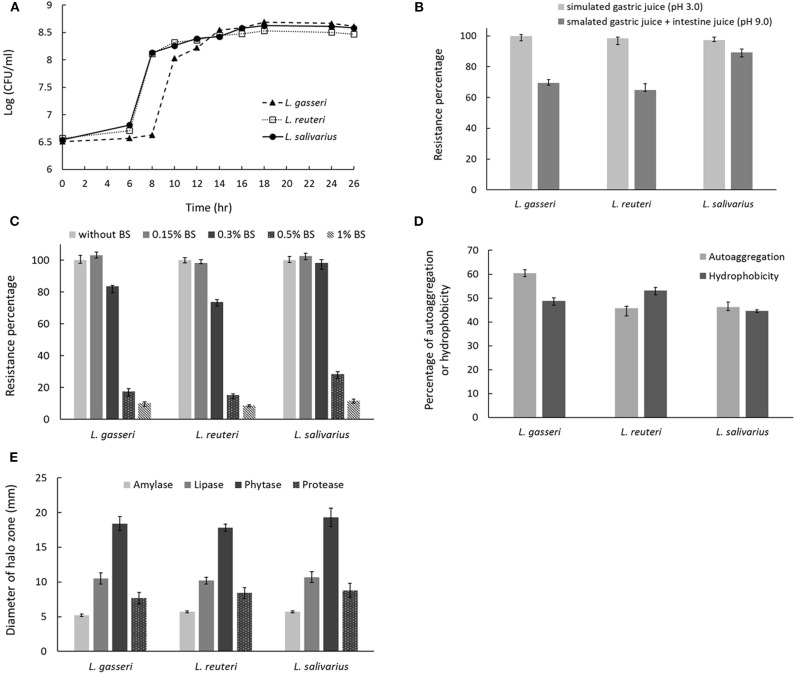
Characterization of probiotic properties of *Lactobacillus* strains. **(A)** The growth curves for the three *Lactobacillus* strains in MRS broth. The effect of incubation in **(B)** simulated gastric and intestinal juices or **(C)** bile salts on cell viability. **(D)** Evaluation of strain autoaggregation propensity and surface hydrophobicity. **(E)** Measurement of digestive enzyme activities, including amylase, lipase, phytase, and protease by spot inoculation method using different agar medium. Enzymatic activity was determined by measuring the diameter of clearing zone surrounding the colonies.

The growth curves of three isolates were shown in Figure 3A. *Lactobacillus reuteri* showed a nearly overlapping growth curve with *L. salivarius*. However, *L. gasseri* exhibited a 2-h delay to reach the start of exponential phase if compared with that of *L. reuteri* or *L. salivarius*. For simulated gastric and small intestine juices tolerance measurement, all the three *Lactobacillus* isolates were enabled to survive at pH 3.0 and 8.0. Among the selected isolates, *L. salivarius* showed superior survival rates of 97.1% and 89.3% to endure the acidic (pH 3.0) and basic (pH 9.0) environment, respectively (Figure 3B). Three *Lactobacillus* isolates showed good survival to 0.3% BSs in MRS broth, whereas BSs up to 0.5% dramatically inhibit the growth of all the isolates (Figure 3C). Determination of cell surface hydrophobicity was evaluated based on the ability of the microorganisms to partition into hydrocarbon from phosphate buffer solution. As shown in Figure 3D, three isolates possessed nearly the same properties in autoaggregation and hydrophobicity, both of which were superior to 45%. The result indicated that three *Lactobacillus* isolates possess great autoaggregative and hydrophobic characteristics in facilitating the adhesion on host epithelial cells. To examine whether three lactobacillus isolates were able to produce active dietary enzymes, the three isolates were assayed for halo formation on agar plates containing certain ingredients. Specific enzyme activity was proportional to the surrounding halo zone sizes. All of the isolates were shown to possess amylase (weak), lipase, phytase, and protease activities (Figure 3E). Maximum halo zone was attributed to *L. salivarius* for lipase (10.7 mm), phytase (19.3 mm), and protease (8.8 mm).

### *Ex vivo* Adhesion to Intestinal Epithelium

**Figure 4:**
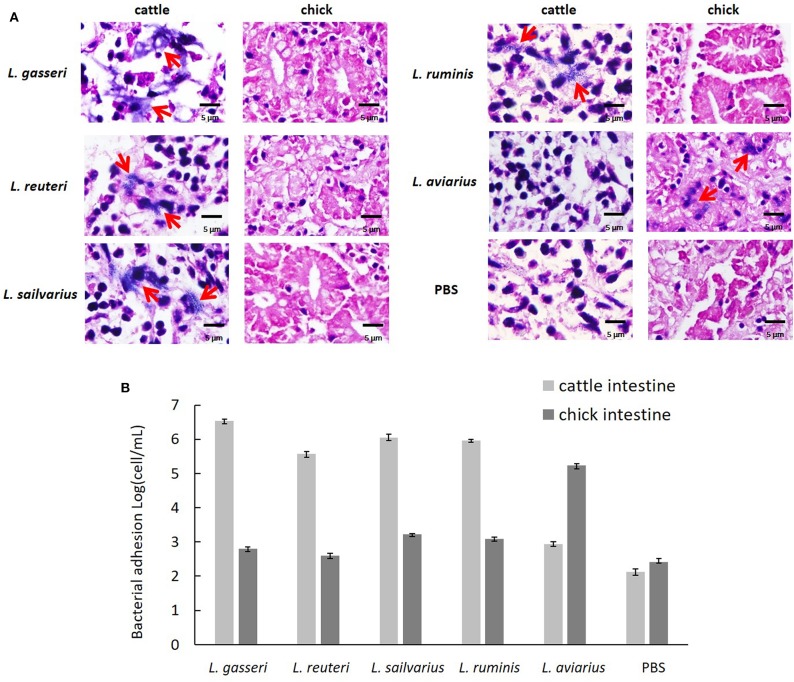
*Ex vivo* adhesion of the *Lactobacillus* strains to intestinal epithelial cells. **(A)** Adhesion of cattle ileum and chick intestine was examined using light microscopy (×400) after Gram staining. Red arrowhead indicates adhered bacteria. **(B)** Quantification of bacteria adherent to intestinal epithelial cells by the Neubauer counting chamber using a light microscope. *Lactobacillus ruminis* and *L. aviarius* isolated, respectively, from bovine rumen and chicken feces served as reference strains. Phosphate-buffered saline was used as a negative control.

The three isolates exhibited a high level of *ex vivo* adhesion to cells in cattle intestinal epithelium when compared to the PBS control (Figure 4A). No obvious adhesion was visible to chick intestinal epithelial cells among the three isolates and *L. ruminis*. In addition, *L. aviaries* adhered specifically to chick intestinal epithelial cells. The number of adhering bacteria was quantified by the Neubauer counting chamber using a light microscope (Figure 4B). The result showed that the three cow-derived isolates specifically adhered to cattle ileal epithelial cells.

### Antagonistic Activity Against Intestinal Pathogens of Cattle

**Figure 5:**
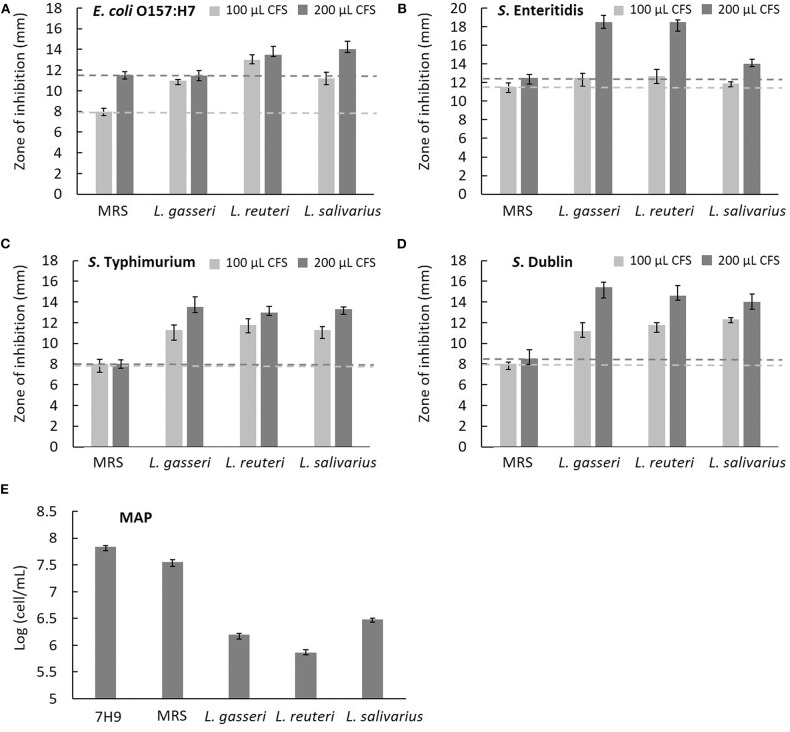
Characterization of the antimicrobial activity of three isolated *Lactobacillus* strains. The cell-free supernatants (CFSs) of *L. gasseri, L. reuteri, L. salivarius*, and acidified MRS (negative control) in the volume of 100 μL (light gray bar) and 200 μL (dark gray bar) were tested for the inhibition of intestinal pathogens of bovine, including **(A)** *E. coli* O157:H7, **(B)** *S. enteritidis*, **(C)** *S. typhimurium*, and **(D)** *S*. Dublin using Oxford Cup methods. The horizontal reference lines (light gray and dark gray dotted lines) represent the ZOI of MRS broth at 100 and 200 μL, respectively. **(E)** The CFS of three isolates (0.5 mL) was tested for the inhibition of MAP growth. Both acidified MRS (0.5 mL at pH 4.0) and 7H9 broth (0.5 mL at pH 6.6) served as references of comparison. *Mycobacterium avium* subspecies *paratuberculosis* cells were counted in a Neubauer chamber at day 28.

The effects of CFS secreted by *Lactobacillus* on the growth of five cattle gut pathogens, including *E. coli* O157:H7, *S. enteritidis, S. typhimurium, S*. Dublin, and MAP, were evaluated by measuring the diameter of the zone of inhibition (ZOI) under Oxford cup diffusion method or using a Neubauer counting chamber. Because most pathogens fail to grow optimally in a lower pH environment, acidified MRS broth (pH 4.0) was used as a reference for comparison. All CFSs of *Lactobacillus* isolates showed distinct inhibitory activity against all the strains of bacteria. Moreover, the inhibitory ability was positively correlated to the volume of CFS (100 and 200 μL). Cell-free supernatants of *L. reuteri* and *L. salivarius* (200 μL) inhibited the growth of *E. coli* O157:H7, which generated a ZOI of 14 mm on the agar plates (Figure 5A). Cell-free supernatants of *L. gasseri* and *L. reuteri* exhibited outstanding inhibitory activity against *S. enteritidis* (ZOI >18 mm at 200 μL CFS; Figure 5B). Three isolates showed similar inhibition ability against *S. typhimurium* (ZOI > 13 mm at 200 μL CFS; Figure 5C). Meanwhile, *L. gasseri, L. reuteri*, and *L. salivarius* showed the significant inhibition to *S*. Dublin, with a ZOI of 15.4, 14.6, and 14.0 mm at 200 μL CFS, respectively (Figure 5D). Because MAP is a fastidious and slow-growing organism, variation in the media (7H9 broth, pH at 6.6) might result in detrimental effects on its growth. In order to clarify the inhibitory effects on pH or ingredients of *Lactobacillus* culture medium, the equal volume (0.5 mL) of acidified MRS broth (pH 4.0) served as a reference in the assay. As shown in Figure 5E, MRS broth caused a slight inhibition of MAP growth. Cell-free supernatants of *Lactobacillus* isolates displayed antagonistic activity against MAP growth, whereas *L. reuteri* possessed the most potent inhibitory activity against MAP (a 2-log reduction). pH values of CFS of *L. gasseri, L. reuteri*, and *L. salivarius* were determined as 4.54 ± 0.02, 4.47 ± 0.02, and 4.03 ± 0.01, respectively (Table 3), implying that the ingredient of CFS might contribute to the antimicrobial potency.

**Table 3:** pH value of cell-free supernatants from *Lactobacillus* strains and reference.

	***L. gasseri***	***L. reuteri***	***L. salivarius***	**Acidified MRS**
pH^a^	4.54 ± 0.02	4.47 ± 0.02	4.03 ± 0.01	4.00 ± 0.02

a *pH value was determined from the bacterial culture after a 16-h growth period in 5 mL MRS broth*.

### Antibiotic Resistance Profile of *Lactobacillus* Isolates

The antimicrobial susceptibility of the three *Lactobacillus* strains was assessed and categorized as either susceptible (S) or resistant (R) based on the breakpoints in the EFSA guideline (Table 4). All tested strains were susceptible to ampicillin (MIC, 1 μg/mL), gentamycin (MIC range, 8–16 μg/mL), erythromycin (MIC, 0.5 μg/mL), clindamycin (MIC, 1 μg/mL), tetracycline (MIC, 4 μg/mL), and chloramphenicol (MIC range, 1–2 μg/mL). *Lactobacillus gasseri* and *L. salivarius* were resistant to streptomycin (MIC, 128 μg/mL) and kanamycin (MIC > 128 μg/mL), respectively. Vancomycin MIC in *L. gasseri* was low (MIC, 2 μg/mL) but was extremely high in *L. reuteri* and *L. salivarius* (MIC >256 μg/mL); however, assessment of vancomycin MIC is not an essential criterion for evaluating probiotic properties of *L. reuteri* and *L. salivarius* (14).

**Table 4:** Antibiotics resistance profiles of the *Lactobacillus* isolates and the respective minimum inhibitory concentration (MIC).

	**MIC (μg/mL)/characterization^a^**		
	***L. gasseri***	***L. reuteri***	***L. salivarius***
Ampicillin	1/S	1/S	1/S
Vancomycin	2/S	>256/n.r.	>256/n.r.
Gentamicin	16/S	8/S	16/S
Kanamycin	64/S	64/S	>256/R
Streptomycin	128/R	64/S	16/S
Erythromycin	0.5/S	0.5/S	0.5/S
Clindamycin	1/S	1/S	1/S
Tetracycline	4/S	4/S	4/S
Chloramphenicol	2/S	1/S	1/S

a *Compared with microbiological breakpoints defined by European Food Safety Authority (EFSA)*.

*S, susceptible; I, intermediate; R, resistant; n.r., not required*.

### Detection and Transfer of Antibiotic Resistance Genes

**Figure 6:**
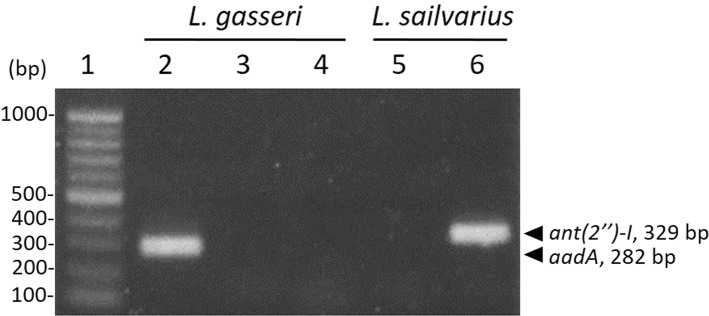
Detection of antibiotic-resistance genes in *Lactobacillus* strains by PCR. Identification of streptomycin (lanes 2–4) and kanamycin (lanes 5 and 6) determinants in *L. gasseri* and *L. salivarius*, respectively. Lane 1, 100-bp DNA ladder; representative *L. gasseri* culture containing *aadA* (lane 2), *aadE* (lane 3), and *ant(6)* (lane 4); representative *L. salivarius* containing *aph(3*″*)-III* (lane 5); *ant(2*″*)-I* (lane 6).

According to the MIC results (Table 4), *L. gasseri* and *L. salivarius* were resistant to streptomycin and kanamycin, respectively. With the widespread use of antibiotics, there are growing concerns regarding bacterial resistance and transfer of antibiotic-resistance genes. Genes that relate to genes of streptomycin resistance [*aadA, aadE*, and *ant(6)*] and kanamycin resistance [*aph(3*″*)-III* and *ant(2*″*)-I*] were examined by PCR using primer sets listed in Table 2. The *aadA* (282 bp) and *ant(2*″*)-I* (329 bp) genes were detectable in *L. gasseri* and *L. salivarius* with expected amplicon sizes (Figure 6). For further evaluation of the transfer potential of antibiotic-resistance genes in *Lactobacillus isolates*, filter mating method was applied using *E. faecalis* JH2-2 as a recipient strain. No colonies of presumptive *aadA* or *ant(2*″*)-I* transconjugants were observed on the selective plates after the mating (Table 5). The data indicate that the *aadA* of *L. gasseri* or *ant(2*″*)-I* of *L. salivarius* was not transferred to recipient strains.

**Table 5:** Calculation of conjugation efficiency.

**Strain**	**Transconjugants (cfu/mL)**	**Recipient (cfu/mL)**	**Conjugation efficiency^a^**
*L. gasseri*	0	7 × 10^9^	0
*L. salivarius*	0	7 × 10^9^	0
*S*. Choleraesuis^b^	1 × 10^3^	7 × 10^9^	1.4 × 10^−7^

a *Conjugation efficiency = number of transconjugants (cfu/mL)/number of recipients (cfu/mL)*.

b *Liao et al. (17)*.

## Discussion

Probiotics maintain normal gut microbiota by competitive exclusion and antagonistic interaction through secretion of antibacterial compounds against pathogens. Early colonization of LAB in the intestinal microflora is likely to interfere with the adherence of pathogens to the intestinal mucosa. Before their colonization of host through mucosal adhesion, probiotics must survive in extremely acidic environment and also must be resistant to BS in the duodenum. Tolerance to insults by digestive chemicals enhances the survival probability of an exogenous culture in the GIT and is therefore criterion for probiotic strain selection (19). *Lactobacillus* species often show superior resistance to gastric juice at pH 3.0, intestine juice, and 0.3% bile, which is an indispensable property for effective probiotics (20). *Lactobacillus salivarius* has been found in both this study and previous studies to be the most tolerant to not only simulated gastric and intestinal juices but also to BS (21).

Evaluation of probiotic surface properties, such as autoaggregation and hydrophobicity, is a prerequisite for selection of effective probiotic bacteria. Autoaggregation is the ability of a bacterial strain to self-interact and correlate with adhesion and GIT colonization efficiency. Cell surface hydrophobicity affects physical and chemical properties of bacterial cells and contributes to adhesion of bacterial cells to host intestinal epithelium cells. A higher hydrophobicity indicates the presence of hydrophobic components on or embedded in the bacterial surface (22). Generally, probiotic strains with hydrophobicity more than 40% were assumed to have greater host adhesion capability and better competitive inhibition of pathogens (23). Moreover, probiotics with extracellular enzyme activity can increase nutrient digestion, a synergistic attribute of benefit to the host. *Lactobacillus* strains isolated from swine intestinal tract harbor amylase, lipase, phytase, and protease activities (24), but are lipase-negative and show variability in amylase and protease activities if isolated from poultry (25).

Host species specificity of probiotic LAB is associated with *ex vivo* adhesion abilities (26) and should be considered as a selection criterion. Factors such as carbohydrate utilization, adhesive structures, and surface proteins that contribute to host adaptation and colonization have been correlated with host specificity of bacterial pathogens (27). Providing species-specific probiotic bacteria supplements promotes animal fitness by fostering a healthy balance between beneficial and pathogenic bacteria and by enhancing host growth, immune status, and nutrient digestion (28).

Antibiotic resistance is an advantageous characteristic for probiotics and provides an opportunity for antimicrobials and strains of complementary resistance to be coadministered as a course of disease treatment. Lactobacilli are intrinsically resistant to several antibiotics and can be beneficial adjunct supplements during antibiotic treatment of GIT conditions (29). The advantages of antibiotic resistance in probiotic LAB strains are coupled with a concern over lateral transfer of antibiotic-resistance genes to other, potentially harmful, gut bacteria. The current study suggests that major antibiotic-resistance genes are unlikely to be horizontally transferred from LAB to recipient bacteria. Administration of these robust LAB strains as a combination of probiotics provides a survival advantage in hosts.

Antimicrobials have long been used to prevent calf scours; however, people have begun to pay an increased amount of attention to safety concerns associated with overuse of antimicrobials, such as antibiotic resistance and persistence of antibiotic residues in animal products. As an alternative to antibiotic overuse, probiotic additives are being developed to deter the spread of pathogenic bacteria and support livestock health. The bacterial strain, *Dietzia* subspecies C79793-74, was initially isolated from paratuberculosis seropositive and fecal-positive cow feces and was subsequently utilized as a therapeutic probiotic for adult paratuberculosis-positive animals (30). For cows with early-stage Johne disease, administration of the probiotic *Dietzia* strain effectively increased animal survival rates with certain cases resulting in cure. *Lactobacillus animalis* NP51 is a bacterium of interest for the food industry because of its ability not only to reduce *E. coli* O157 and *Salmonella* species shedding but also to reduce immuno-mediated chronic inflammation associated with MAP infection (31). *Lactobacillus acidophilus, Lactobacillus curvatus* TUCO-5E, and *Lactobacillus plantarum* are host-specific probiotics that possess antimicrobial activity against *E. coli, Enterobacter* species, and *Salmonella* in swine (6).

*Lactobacillus gasseri*, common to the microbial flora of both the human GIT and vagina, produces lactocillin (a thiopeptide antibiotic) and gassericin A (a bacteriocin) and activates macrophages (32) against potentially pathogenic organisms. In addition, *L. gasseri* isolated from the vaginal tract of cattle has been shown to inhibit *Staphylococcus aureus* through lactic acid and hydrogen peroxide production (33). *Lactobacillus reuteri* is mainly isolated from the jejunum, ileum, and rectum of mammals. The beneficial effects of *L. reuteri* include the production of antimicrobial molecules (organic acids, ethanol, and reuterin), the inhibition of pathogenic microbe colonization and the remodeling of the commensal microbiota composition in the host (34). *Lactobacillus reuteri* promotes regulatory T-cell differentiation and decreases proinflammatory cytokine production, thereby serving as a promising therapy against inflammatory diseases (35). Recently, *L. reuteri* LB1-7, a strain derived from raw cow milk, was shown to possess antimicrobial activity against an O157:H7 enterohemorrhagic *E. coli* strain via producing hydroxypropionaldehyde under strict anaerobiosis (36). *Lactobacillus salivarius* has been found to live in the GIT and benefits in therapeutic properties including suppression of pathogenic bacteria by producing organic acids, hydrogen peroxide, and other antimicrobial substances (37).

In this study, bovine-specific fecal-derived probiotic lactobacilli candidates were isolated using valid selection criteria. All the isolates possessed superior probiotic attributes such as resistance to BSs and low pH, a high hydrophobicity, and nutrient-freeing digestive enzyme activities. Moreover, the LAB isolates possess the ability to inhibit the growth of several foodborne pathogens including *E. coli* O157:H7, MAP, *S. enteritidis, S. typhimurium*, and *S*. Dublin. Host specificity demonstrated by *ex vivo* adhesion toward bovine intestinal epithelial cells was also observed. Because isolation of probiotics from the native host might provide a better adaptation to its original habitat, the newly identified *Lactobacillus* strains have potential to serve as species-specific probiotics with a low risk of lateral transfer of the antibiotic-resistance genes for cows. Future *in vivo* studies using oral administration of the strains are required to assess their effectiveness in dairy cows as biotherapeutic agents and antibiotic alternatives.

## Data Availability Statement

The sequence data in this study has been deposited to the GenBank database (https://www.ncbi.nlm.nih.gov/genbank/) using the accession numbers: MT268541, MT268630, MT268707.

## Ethics Statement

This study was conducted under ethical approval from the Institutional Animal Care and Use Committee (IACUC) of National Chung Hsing University (IACUC permit number 107-045).

## Author Contributions

W-CL, C-YC, M-KI, and C-JK conducted the experiments and analyzed data. M-YC and T-HC helped in conception and design of the study. CP and C-JK designed the experiment and wrote the manuscript.

## Conflict of Interest

The authors declare that the research was conducted in the absence of any commercial or financial relationships that could be construed as a potential conflict of interest.
