# Supplementary material for: Autochthonous Lactic Acid Bacteria Isolated From Dairy Cow Feces Exhibiting Promising Probiotic Properties and in vitro Antibacterial Activity Against Foodborne Pathogens in Cattle
Source: Front Vet Sci. 2020 May 15;7:239. doi: 10.3389/fvets.2020.00239 (PMC7243249; doi:10.3389/fvets.2020.00239)
Supplement: Supplementary file 1 [file Data_Sheet_1.docx]

Supplementary material

16S rDNA sequence result of *L. gasseri*

GAAGGAAAATGAGAGTTTGATCCTGGCTCAGGACGAACGCTGGCGGCGTGCCTAATACATGCAAGTCGAGCGAGCTTGCCTAGATGAATTTGGTGCTTGCACCAGATGAAACTAGATACAAGCGAGCGGCGGACGGGTGAGTAACACGTGGGTAACCTGCCCAAGAGACTGGGATAACACCTGGAAACAGATGCTAATACCGGATAACAACACTAGACGCATGTCTAGAGTTTAAAAGATGGTTCTGCTATCACTCTTGGATGGACCTGCGGTGCATTAGCTAGTTGGTAAGGTAACGGCTTACCAAGGCAATGATGCATAGCCGAGTTGAGAGACTGATCGGCCACATTGGGACTGAGACACGGCCCAAACTCCTACGGGAGGCAGCAGTAGGGAATCTTCCACAATGGACGCAAGTCTGATGGAGCAACGCCGCGTGAGTGAAGAAGGGTTTCGGCTCGTAAAGCTCTGTTGGTAGTGAAGAAAGATAGAGGTAGTAACTGGCCTTTATTTGACGGTAATTACTTAGAAAGTCACGGCTAACTACGTGCCAGCAGCCGCGGTAATACGTAGGTGGCAAGCGTTGTCCGGATTTATTGGGCGTAAAGCGAGTGCAGGCGGTTCAATAAGTCTGATGTGAAAGCCTTCGGCTCAACCGGAGAATTGCATCAGAAACTGTTGAACTTGAGTGCAGAAGAGGAGAGTGGAACTCCATGTGTAGCGGTGGAATGCGTAGATATATGGAAGAACACCAGTGGCGAAGGCGGCTCTCTGGTCTGCAACTGACGCTGAGGCTCGAAAGCATGGGTAGCGAACAGGATTAGATACCCTGGTAGTCCATGCCGTAAACGATGAGTGCTAAGTGTTGGGAGGTTTCCGCCTCTCAGTGCTGCAGCTAACGCATTAAGCACTCCGCCTGGGGAGTACGACCGCAAGGTTGAAACTCAAAGGAATTGACGGGGGCCCGCACAAGCGGTGGAGCATGTGGTTTAATTCGAAGCAACGCGAAGAACCTTACCAGGTCTTGACATCCAGTGCAAACCTAAGAGATTAGGTGTTCCCTTCGGGGACGCTGAGACAGGTGGTGCATGGCTGTCGTCAGCTCGTGTCGTGAGATGTTGGGTTAAGTCCCGCAACGAGCGCAACCCTTGTCATTAGTTGCCATCATTAAGTTGGGCACTCTAATGAGACTGCCGGTGACAAACCGGAGGAAGGTGGGGATGACGTCAAGTCATCATGCCCCTTATGACCTGGGCTACACACGTGCTACAATGGACGGTACAACGAGAAGCGAACCTGCGAAGGCAAGCGGATCTCTGAAAGCCGTTCTCAGTTCGGACTGTAGGCTGCAACTCGCCTACACGAAGCTGGAATCGCTAGTAATCGCGGATCAGCACGCCGCGGTGAATACGTTCCCGGGCCTTGTACACACCGCCCGTCACACCATGAGAGTCTGTAACACCCAAAGCCGGTGGGATAACCTTTATAGGAGTCAGCCGTCTAAGGTAGGACAGATGATTAGGGTGAAGTCGTAACAAGGTAGCCGTAGGAGAACCTGCGGCTGGATCACCTCCTTTCT

16S rDNA sequence result of *L. reuteri*

GTGCCTAATACATGCAAGTCGTACGCACTGGCCCAACTGATTGATGGTGCTTGCACCTGATTGACGATGGATCACCAGTGAGTGGCGGACGGGTGAGTAACACGTAGGTAACCTGCCCCGGAGCGGGGGATAACATTTGGAAACAGATGCTAATACCGCATAACAACAAAAGCCGCATGGCTTTTGTTTGAAAGATGGCTTTGGCTATCACTCTGGGATGGACCTGCGGTGCATTAGCTAGTTGGTAAGGTAACGGCTTACCAAGGCGATGATGCATAGCCGAGTTGAGAGACTGATCGGCCACAATGGAACTGAGACACGGTCCATACTCCTACGGGAGGCAGCAGTAGGGAATCTTCCACAATGGGCGCAAGCCTGATGGAGCAACACCGCGTGAGTGAAGAAGGGTTTCGGCTCGTAAAGCTCTGTTGTTGGAGAAGAACGTGCGTGAGAGTAACTGTTCACGCAGTGACGGTATCCAACCAGAAAGTCACGGCTAACTACGTGCCAGCAGCCGCGGTAATACGTAGGTGGCAAGCGTTATCCGGATTTATTGGGCGTAAAGCGAGCGCAGGCGGTTGCTTAGGTCTGATGTGAAAGCCTTCGGCTTAACCGAAGAAGTGCATCGGAAACCGGGCGACTTGAGTGCAGAAGAGGACAGTGGAACTCCATGTGTAGCGGTGGAATGCGTAGATATATGGAAGAACACCAGTGGCGAAGGCGGCTGTCTGGTCTGCAACTGACGCTGAGGCTCGAAAGCATGGGTAGCGAACAGGATTAGATACCCTGGTAGTCCATGCCGTAAACGATGAGTGCTAGGTGTTGGAGGGTTTCCGCCCTTCAGTGCCGGAGCTAACGCATTAAGCACTCCGCCTGGGGAGTACGACCGCAAGGTTGAAACTCAAAGGAATTGACGGGGGCCCGCACAAGCGGTGGAGCATGTGGTTTAATTCGAAGCTACGCGAAGAACCTTACCAGGTCTTGACATCTTGCGCTAACCTTAGAGATAAGGCGTTCCCTTCGGGGACGCAATGACAGGTGGTGCATGGTCGTCGTCAGCTCGTGTCGTGAGATGTTGGGTTAAGTCCCGCAACGAGCGCAACCCTTGTTACTAGTTGCCAGCATTAAGTTGGGCACTCTAGTGAGACTGCCGGTGACAAACCGGAGGAAGGTGGGGACGACGTCAGATCATCATGCCCCTTATGACCTGGGCTACACACGTGCTACAATGGACGGTACAACGAGTCGCAAACTCGCGAGAGTAAGCTAATCTCTTAAAGCCGTTCTCAGTTCGGACTGTAGGCTGCAACTCGCCTACACGAAGTCGGAATCGCTAGTAATCGCGGATCAGCATGCCGCGGTGAATACGTTCCCGGGCCTTGTACACACCGCCCGTCACACCATGGGAGTTTGTAACGCCCAAAGTCGGTGGCCTAACCTTTATGGAGGGAGCCGCCTAAGGCGGACAGA

16S rDNA sequence result of *L. salivarius*

AACGCTGGCGGCGTGCCTATACATGCAAGTCGAACGAAACTTTCTTACACCGAATGCTTGCATTCATCGTAAGAAGTTGAGTGGCGGACGGGTGAGTAACACGTGGGTAACCTGCCTAAAAGAAGGGGATAACACTTGGAAACAGGTGCTAATACCGTATATCTCTAAGGATCGCATGATCCTTAGATGAAAGATGGTTCTGCTATCGCTTTTAGATGGACCCGCGGCGTATTAACTAGTTGGTGGGGTAACGGCCTACCAAGGTGATGATACGTAGCCGAACTGAGAGGTTGATCGGCCACATTGGGACTGAGACACGGCCCAAACTCCTACGGGAGGCAGCAGTAGGGAATCTTCCACAATGGACGCAAGTCTGATGGAGCAACGCCGCGTGAGTGAAGAAGGTCTTCGGATCGTAAAACTCTGTTGTTAGAGAAGAACACGAGTGAGAGTAACTGTTCATTCGATGACGGTATCTAACCAGCAAGTCACGGCTAACTACGTGCCAGCAGCCGCGGTAATACGTAGGTGGCAAGCGTTGTCCGGATTTATTGGGCGTAAAGGGAACGCAGGCGGTCTTTTAAGTCTGATGTGAAAGCCTTCGGCTTAACCGGAGTAGTGCATTGGAAACTGGAAGACTTGAGTGCAGAAGAGGAGAGTGGAACTCCATGTGTAGCGGTGAAATGCGTAGATATATGGAAGAACACCAGTGGCGAAAGCGGCTCTCTGGTCTGTAACTGACGCTGAGGTTCGAAAGCGTGGGTAGCAAACAGGATTAGATACCCTGGTAGTCCACGCCGTAAACGATGAATGCTAGGTGTTGGAGGGTTTCCGCCCTTCAGTGCCGCAGCTAACGCAATAAGCATTCCGCCTGGGGAGTACGACCGCAAGGTTGAAACTCAAAGGAATTGACGGGGGCCCGCACAAGCGGTGGAGCATGTGGTTTAATTCGAAGCAACGCGAAGAACCTTACCAGGTCTTGACATCCTTTGACCACCTAAGAGATTAGGCTTTCCCTTCGGGGACAAAGTGACAGGTGGTGCATGGCTGTCGTCAGCTCGTGTCGTGAGATGTTGGGTTAAGTCCCGCAACGAGCGCAACCCTTGTTGTCAGTTGCCAGCATTAAGTTGGGCACTCTGGCGAGACTGCCGGTGACAAACCGGAGGAAGGTGGGGACGACGTCAAGTCATCATGCCCCTTATGACCTGGGCTACACACGTGCTACAATGGACGGTACAACGAGTCGCGAGACCGCGAGGTTTAGCTAATCTCTTAAAGCCGTTCTCAGTTCGGATTGTAGGCTGCAACTCGCCTACATGAAGTCGGAATCGCTAGTAATCGCGAATCAGCATGTCGCGGTGAATACGTTCCCGGGCCTTGTACACACCGCCCGTCACACCATGAGAGTTTGTAACACCCAAAGCCGGTGGGGTAACCGCAAGGAGCCAGCCGTCTAAGGTGGACAGAT
